# Leading by example: a migrant health researcher’s journey from participation to epistemic justice and migrant-centred governance in public health research

**DOI:** 10.1186/s12939-026-03055-y

**Published:** 2026-10-14

**Authors:** Maria Gabriela Uribe Guajardo

**Affiliations:** The Leeder Centre for Health Policy, Economics and Data, Sydney School of Public Health, University of Sydney, Sydney, Australia

**Keywords:** Migrant researcher, Public health, Epistemic justice, Migrant-centred governance

## Abstract

Participatory and co-produced approaches are increasingly promoted in migrant health research to improve equity and ensure research reflects community priorities. Despite growing guidance on conducting research with migrants, migrants remain underrepresented in research leadership, governance, agenda-setting, and knowledge production. Drawing on lived experience as both a migrant and public health researcher, this reflective paper examines the evolution of a program of research spanning health and social care integration, public health emergency preparedness, and international collaborations with migrant communities, frontline workers, and researchers. The paper traces a progression from a focus on service responsiveness and integration towards broader questions of participation, power-sharing, and knowledge justice. Findings highlight the importance of sustained relationships, and community partnerships in generating meaningful and actionable evidence. The work demonstrates how recognising migrants as legitimate knowledge holders through shared governance, remuneration, authorship, and leadership roles can strengthen both the quality and relevance of research. These experiences illustrate a shift from consultation and collaboration towards migrant-led approaches to knowledge generation. The next stage of migrant health research should move beyond participation and co-production towards epistemic justice and migrant-centred governance. This requires challenging traditional hierarchies of expertise and recognising migrants not only as research participants but as leaders, agenda-setters, and producers of knowledge. Such approaches offer a pathway towards more equitable, accountable, and responsive public health research, policy, and practice.

## Background

Public health research increasingly emphasises community engagement, participation, and co-production as mechanisms for improving equity and ensuring knowledge reflect the needs of diverse populations [1]. Participatory approaches have become particularly prominent in-migrant health research, where they are viewed as essential for developing research and services that are responsive to the needs, strengths, and experiences of migrant and ethnic diverse communities [2].

A plethora of frameworks on ‘how to do’ research ‘with’ migrants have been developed [1–3]. These elucidates the value of involving refugees and migrants in research (including service design) and research agendas and the need to develop an ecosystem that will support and sustain participatory, interdisciplinary and inter-sectoral projects.

While participatory and co-produced approaches are now well established within migrant health research, and a growing literature examines power, authorship, and decolonising knowledge production [4], real-world stories of co-creation are inconsistently reported and often concentrated in operational aspects of projects rather than in agenda-setting, analysis, leadership, dissemination, or decision-making [5–7]. Research continues to be conducted predominantly on migrants rather than with migrants as equal partners in knowledge production [8].

As researchers, funders, and institutions increasingly promote community engagement and participatory approaches, there is growing recognition that migrants should be involved from the earliest stages of research, including identifying priorities and shaping research agendas [8]. Participatory health research frameworks emphasise migrants’ involvement in defining research questions and co-creating solutions to health challenges. However, while there is a growing body of guidance on conducting research with migrants, there remains limited scholarship documenting how migrants move from community engagement and participation towards becoming leaders of knowledge generation, agenda setters, and recognised producers of public health evidence [8].

In this paper, I reflect on how lived experience of migration informed my progression from community engagement and advocacy to becoming a public health researcher who intentionally engages migrant leaders as experts and co-producers of knowledge. I argue that the next stage of migrant health research should move beyond participation and co-production towards recognising of individuals and communities as legitimate knowers whose knowledge is valued, and incorporated into decision-making systematically. In public health, this requires challenging traditional hierarchies that privilege institutional and academic expertise while marginalising lived expertise.

Specifically, this paper aims to critically reflect on an evolving program of migrant health research and argue for a shift from participation to epistemic justice in public health. Epistemic justice is all about allowing or enabling marginalised researchers and communities to think about and analyse their experiences in ways that value and appropriately recognise those experiences, and particularly so when these clash with the perspectives of the dominantly situated and hegemonic discourses [9]. Without such recognition, existing patterns of epistemic marginalisation are likely to be reinforced rather than challenged [9].

Using examples from health and social care integration research [10–12], community-engaged public health emergency preparedness research [13, 14], and international collaborations with researchers, migrant researchers and migrant frontline workers [15, 16], this work examines how approaches to engagement evolved from consultation to collaboration, ultimately, towards migrant-led shared governance.

## Main text

### Positionality

I have occupied a dual position as both a scholar and a migrant who has navigated health, social, educational, and institutional systems in a new country [16]. This experience has shaped the research questions and the way my program of research works with community, building partnerships and create knowledge.

This academic fellowship, although has some traditional roots (e.g. medical funding, being part of an academic institution), has allowed me and encourage to explore, using qualitative methods, and participatory approaches the topic of migrant participation in the context of integrated health and social care with both lenses, academic scholarship and new models of thinking derived from lived experience. This work reflects on my pathway moving from how migrants are represented in service integration research to shared knowledge generation.

**Fig. 1:**
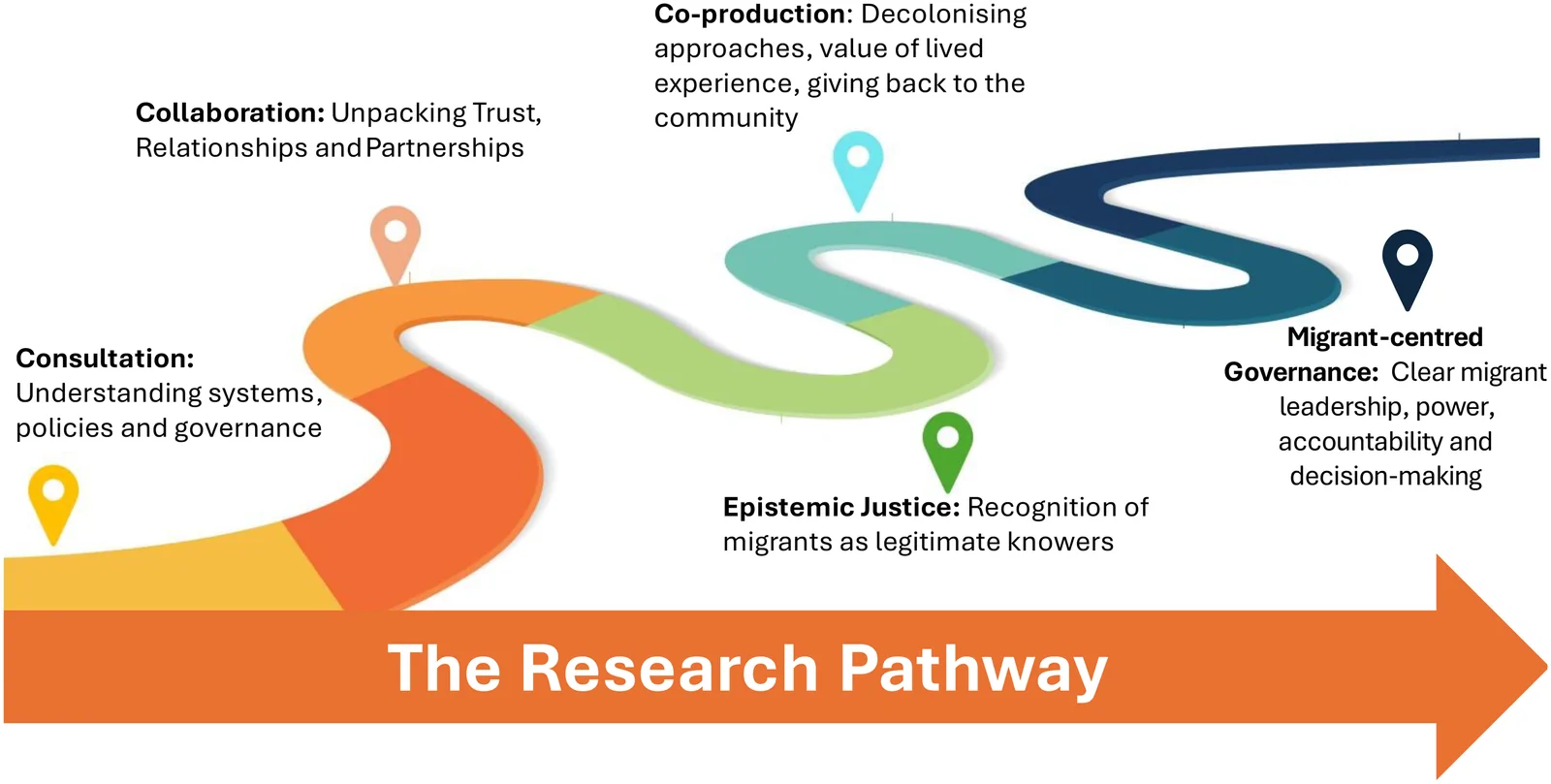
The evolution of research towards migrant-centred governance

Figure 1 illustrates the evolution of the author’s research program from service responsiveness and consultation and collaboration towards co-production, epistemic justice and recognition of migrants as legitimate knowledge holders, and ultimately migrant-centred governance.

### Migrants’ voices and its place in service provision and research

The first stage of my migrant researcher journey was focused on the understanding and strengthening the systems that support migrants before shifting attention to who holds power within those systems [11].

Specifically, I examined the key policy mechanisms required to improve integration between health and social care systems for migrant communities in Sydney, Australia [11]. The outputs identified how governance arrangements, partnerships, workforce models and service coordination can support more integrated responses to migrant health and social needs, while highlighting persistent fragmentation across sectors [11]. Although existing models have made some progress, there is a clear lack of community representatives (migrant themselves) formally embedded in health or social care systems impacting research, policy and practice.

Further evidence identifying data sharing between sectors (and information technologies) is limited, demonstrating the lack of effective information exchange, governance and funding structures and organisational capacity to support migrant communities’ needs [10, 12].

Collectively, these studies revealed that effective migrant responsiveness depends not only on service delivery, but also on the policy infrastructures that enable organisations to work together through shared governance (including clear embedded community decision-making), information and resources. This early work also revealed that meaningful and equitable integration of migrant voices in research, policy and practice remains low, and researchers are yet to challenge the system and work on more sustainable ways for allowing this to occur.

### Trust, relationships and the invisible work

The relationships and key partnerships created through this research program have provided access to diverse forms of knowledge and expertise that could not have been captured through any single perspective. Policymakers shared insights into system-level priorities and constraints; service providers described the practical realities of implementation; and community-led organisations and migrant volunteers helped illuminate how policies and services are experienced by migrant and culturally diverse communities [11]. Bringing these perspectives together enabled a more comprehensive understanding of the barriers and opportunities for improving health and social care responsiveness for equity with migrants and for migrants [11, 15].

My research has demonstrated the key role that relationships and partnership (formal and informal) played during COVID-19 pandemic and how they were crucial in translating health and public information, facilitating access to health and social services, identifying emerging needs, and supporting those communities [13].

However, meaningful engagement with migrant representatives and voices, in research and practice requires significant investments of time, trust and relationship-building. This is also the case for formal institutions and services who also need to develop trust in community-led organisations and individuals. Establishing partnerships across health and social care and community sectors involved ongoing communication, repeated engagement, coordination of diverse interests and a commitment to understanding interest-holder priorities beyond the immediate objectives of crises, research projects and agendas. While this relational work is ***often invisible*** within traditional measures of academic productivity, it is essential for producing evidence that is relevant, actionable and capable of supporting system-wide change.

Importantly, (I see that) the partnerships developed through this research have therefore been both a *mechanism for generating evidence* and an *outcome (getting individuals and systems together)*, fostering connections that support ongoing collaboration and contribute to more integrated, responsive and inclusive health and social systems.

### Co-production process

My program of work has also been crucial to understand what does ‘co-production mean’ in the context of migrant and social care research. There are key elements to understand ‘co-production’. After acknowledging positionality, and develop trust and relationships with community, creating a space for valuing lived experience and avoid colonising narratives (and frameworks) is paramount.

Co-production often start before ‘co-production’. Engaging and get to know migrants leaders before engaging with a project grant application or research projects have been useful, leveraging from those fully formed relationships before coming together for a project [14].

Recognising communities as legitimate knowledge holders and contributors to knowledge generation (e.g. authors in publications) can create a shift toward epistemic justice and migrant-centred governance [16, 17].

In my research, forming a more formal governance where migrants’ representatives are driving the research formally (including reimbursement) has strengthen the knowledge generation, making sure that the research is not position for epistemic extraction, while making sure you are giving back to the community in ways they chose to define [14].

When co-producing, it is also important to be mindful of not rejecting other narratives just because are not traditional. Rather, research approaches should prioritise and accommodate diverse forms of knowledge, including those grounded in lived experience, culture, and community practice. This may necessitate challenging, adapting, or rejecting colonising frameworks that fail to align with the realities and priorities of participating communities [15].

## Conclusion

This program of research enabled engagement with diverse interest-holders and created the conditions for a progression from questions of service integration [10–13, 18] towards broader concerns of participation, power-sharing, knowledge justice, and ultimately, migrant-centred governance [14, 16].

In this paper, I challenge traditional hierarchies of knowledge production that have historically positioned migrants as research participants, beneficiaries, or subjects of inquiry, rather than as partners in governance and producers of knowledge [16].

Central to this shift is a move away from framing migrants solely through narratives of vulnerability and disadvantage. Although attention to structural inequities remains essential, shared governance requires a simultaneous recognition of migrant agency, creativity, resilience, resistance and leadership [15].

A further implication of this work is the importance of community-defined priorities and outcomes. Existing governance and research systems frequently rely on institutionally determined indicators of success (e.g. academic outputs), often overlooking what communities themselves value. This challenge traditional models and creates space for communities to participate not only in identifying problems but also in defining desired outcomes, cultural expression, social connectedness, and self-determination [16].

Underlying these principles is the recognition that reciprocity, and sustained engagement are not ancillary to governance but constitute governance itself. Relational infrastructure, including long-term partnership building, shared accountability, and ongoing investment in community relationships, represents a critical yet frequently undervalued component of shared governance. Without such relational foundations, participation risks becoming symbolic rather than transformative.

Collaboration concerns relationships and partnerships; co-production concerns meaningful contributions to research and practice; epistemic justice concerns the recognition of migrants as legitimate knowers; and governance concerns authority, power, and decision-making.

Viewed in this way, the next evolution of migrant health research lies not simply in strengthening participation or co-production, but in advancing models of migrant-centred governance that support epistemic justice in institutions including and beyond academia. Future endeavours should promote both the advancement of sustainable models of migrant-shared governance that are institutionally embedded and the reporting of these processes as part of the outcome of the migrant research.

## Data Availability

No datasets were generated or analysed during the current study.

## References

[1] Roura M, Dias S, LeMaster JW, MacFarlane A. Participatory health research with migrants: Opportunities, challenges, and way forwards. Health Expect. 2021;24(Electronic):1369–7625. 10.1111/hex.13201 PMC8077110 33528082

[2] Giménez L, Evangelidou S, Gresle AS, de la Torre L, Ubalde-López M, Recasens O, et al. Living labs for migrant health research: the challenge of cocreating research with migrant population and policy makers. LID. 10.1136/bmjgh-2023-014795. LID - e014795. (2059–7908 (Print)). PMC11367358 39209337

[3] MacFarlane A, Puthoopparambil SJ, Waagensen E, Sisti LG, Costanzo G, Kayi I, et al. Framework for refugee and migrant health research in the WHO European Region. Trop Med Int Health. 2025(1365–3156 (Electronic)). 10.1111/tmi.13845 PMC10107437 36576432

[4] Tagoe N, Abimbola S, Bilardi D, Kamuya D, Gilson L, Muraya K, et al. Creating different global health futures: mapping the health research ecosystem and taking decolonial action. BMC Health Serv Res. 2025(1472–6963 (Electronic)). 10.1186/s12913-025-12566-3 PMC12007233 40247332

[5] Luetke Lanfer H, Landwehr J, Reifegerste D. Participatory health research and promotion with migrant communities in Germany: a scoping review. Front Public Health. 2025;13. 10.3389/fpubh.2025.1585178 PMC12463829 41018798

[6] Price K, Lenart A, Taknint JT, Gellatly R, Alshabani N. Addressing Barriers To Forcibly Displaced Migrants’ Participation in Research. J Immigr Minor Health. 2026;28(3):747–56. 10.1007/s10903-026-01878-z 41678130

[7] Vazquez Corona M, Hazfiarini A, Vaughan C, Block K, Bohren MA. Participatory Health Research With Women From Refugee, Asylum-Seeker, and Migrant Backgrounds Living in High-Income Countries: A Scoping Review. Int J Qualitative Methods. 2024;23:16094069231225371.

[8] MacFarlane A, Huschke S, Marques MJ, Gama A, Kinaan W, Hassan A, et al. Normalising participatory health research approaches in the WHO European region for refugee and migrant health: a paradigm shift. Lancet Reg Health Eur. 2024(2666–7762 (Electronic)). 10.1016/j.lanepe.2024.100837 PMC11306213 39119099

[9] Landström K, Crawley H. Migration Research, Coloniality and Epistemic Injustice. In: Crawley H, Teye JK, editors. The Palgrave Handbook of South–South Migration and Inequality. Cham: Springer International Publishing; 2024. pp. 83–104.

[10] Uribe Guajardo MG, Giannopoulos V, Jones T, Gillespie J, Cunich M, Eastwood J, et al. Funding models in health and social care services focused on culturally and linguistically diverse populations in Sydney, Australia. J Integr Care. 2026;34(5):1–10.

[11] Uribe Guajardo MG, Mukumbang F, Dronavalli M, Woolfenden S, Parcsi L, McDougall B, et al. Innovative Policy Supports for Integration in Health and Social Care Focused on Culturally and Linguistically Diverse Populations in Australia: A Qualitative Study. J Immigr Minor Health. 2025;27(4):550–8. 10.1007/s10903-025-01697-8 PMC12255537 40299170

[12] Uribe Guajardo MG, Giannopoulos V, Cunich M, Gillespie J, Shaw T, Eastwood J, et al. Data sharing in health and social care in Australia: lessons for integrating care for priority populations. npj Digit Public Health. 2026;1(1):1.

[13] Uribe Guajardo M, Marcus K, Milat A, Cunich M, Eastwood J, Huckel Schneider C, et al. Insights from stakeholders in Sydney’s diverse ethnic communities during the COVID-19 Delta phase. Public Health. 2025;245:105790. 10.1016/j.puhe.2025.105790 40435925

[14] Uribe Guajardo G, Cunich M, Wilson A, Ravi S, Faraj A, Serhan Z, et al. Filling the gap: a new approach to developing a framework for culturally inclusive communication responses during public health emergencies 2026.

[15] Uribe Guajardo M, Diab JL, Curry G, Lamarque M, Mazariego J, Briozzo E, et al. Advancing participatory approaches in global migrant research: a roundtable discussion. J Immigr Minor Health. 2025. 10.1007/s10903-025-01798-4 41152661

[16] Uribe Guajardo G, Curry G, Lamarque M, Mazariego J, Briozzo E, Stojanovski K, et al. Saying the quiet part out loud: The perspectives of migrant frontline workers on participatory research in migration. J. Glob. Health. In press. 2026.

[17] Sam-Agudu NA, Abimbola S. Using scientific authorship criteria as a tool for equitable inclusion in global health research. BMJ Global Health. 2021;6(10):e007632. 10.1136/bmjgh-2021-007632 PMC8506888 34649868

[18] Uribe Guajardo MG, Mukumbang F, Moore C, Jones T, Woolfenden S, Ostojic K, et al. How can we define social care and what are the levels of true integration in integrated care? A narrative review. J Integr Care. 2023.

